# How Reframing Affects Confidence in Complex Decisions: Evidence from Behavioral Measures and Decisional Styles

**DOI:** 10.3390/brainsci15030244

**Published:** 2025-02-25

**Authors:** Michela Balconi, Angelica Daffinà, Laura Angioletti

**Affiliations:** 1 International Research Center for Cognitive Applied Neuroscience (IrcCAN), Università Cattolica del Sacro Cuore, Largo Gemelli 1, 20123 Milan, Italy; michela.balconi@unicatt.it (M.B.);; 2 Research Unit in Affective and Social Neuroscience, Department of Psychology, Università Cattolica del Sacro Cuore, Largo Gemelli 1, 20123 Milan, Italy

**Keywords:** reframe, decision-making, cognitive bias, confidence rating, cognitive load, decision-making style

## Abstract

**Background/Objectives**: This research examined the impact of reframing on decision confidence and its link with individual decision-making styles in a sample of healthy adults. **Methods**: Participants completed a Resistance to Reframe Task, which involved two decision-making steps. In each step, they chose the best option from four alternatives for a workplace situation and rated their confidence in the decision. Then, the task was reframed to highlight the negative consequences of their initial choice, and they reassessed their confidence. Confidence scores and reaction times (RTs) were recorded for the confidence ratings of each step. The General Decision-Making Style (GDMS) and Maximization Scale (MS) were also used to profile decision-making styles and explore their links to behavioral responses. **Results**: Findings demonstrated that reframing significantly reduces participants’ confidence, particularly in the first step, highlighting its effectiveness in challenging initial choices. Additionally, higher RTs after reframing emphasize the cognitive complexity introduced by the change of perspective and allows us to describe the dynamic of the decision-making process. Correlational findings suggested that while some traits (e.g., dependent style) reduce confidence after the reframing, others (e.g., high standards, decision difficulty) seem to reinforce it. Similarly, decision-making style as MS alternative search increases RTs, reflecting the heightened complexity of reframed decision contexts. **Conclusions**: The results underscored the importance of considering confidence in the decision and individual differences when studying decision-making under reframing conditions. Individual differences in decision-making styles may act as protective or vulnerability factors to reframe in decision-making processes.

## 1. Introduction

Is the glass half full or half empty? This timeless question exemplifies how varying the frame and the perspectives on the same issue can shape both the response and the attitude during a decision-making process.

The ability to be flexible in decisions, change one’s outlook, and adapt to changes has been valued as a positive characteristic among expert decision-makers [1]. However, on the other hand, it proves useful for individuals to also be able to maintain one’s decision independently from the context, for instance, despite a situation being subjected to covert influence tactics that act on the context, such as reframing strategies. Indeed, among other covert influence tactics, changing the framework of a situation or question (a strategy known as “reframing”) has been widely demonstrated to influence the outcome of a decision by Tversky and Kahneman [2] as well as more recent studies [3,4].

Originally studied within therapeutic settings, the strategy of reframing relates to a method of interpretation that offers an alternative meaning or viewpoint, steering perspectives in a constructive manner (e.g., positive reframing; [5,6]), frequently deriving beneficial implications from challenging situations. In contrast, negative reframing focuses on presenting the behavioral outcomes in a negative light [7]. It has been applied in research on communication and politics [8,9], and has even extended into the organizational domain, helping professionals view company challenges from diverse perspectives [10,11,12].

In a recent organizational study, we examined how professionals with varying levels of seniority are able to resist contextual biases, specifically reframing and decoy effects, during decision-making processes [13]. Two different decision-making tasks were proposed to the participants: the Resistance to Reframe Task (RRT) and the Resistance to Alternatives Task, involving typical scenarios in the workplace domain. Findings indicated that professionals are generally able to resist reframing scenarios, with no significant differences between senior and junior professionals. Resistance to reframing was operationalized by measuring the change in participants’ decision confidence before and after the introduction of a new perspective or reframing. However, differences in decision-making styles showed to influence the resistance to such bias: for instance, a dependent decision-making style correlates with reduced resistance to these biases, particularly among senior professionals. For junior professionals, both a dependent decision-making style and a tendency to set high standards are linked to decreased resistance to decoy effects. The study concludes that while professionals can maintain consistent decision-making in the face of reframing, certain decision-making styles may increase vulnerability to these biases.

Despite this study being one of the first to focus on resistance to reframing and decision-making styles in a sample of professionals, to our knowledge, similar studies have never been applied to healthy young adults not belonging to a specific population. Furthermore, in a previous work [13], behavioral indices were calculated to assess the overall resistance to reframing of professionals, while the dynamic of the process and the level of confidence in one’s decision (before and after the reframe) were not taken into account.

Therefore, there seems to be a gap in the existing literature regarding the influence of reframing on decision confidence and its relation to decision-making styles.

Indeed, individual differences in decision-making styles play a critical role in how people process reframed information and integrate it into their decision-making processes. Decision-making styles can be viewed as learned tendencies to respond in a particular manner within specific decision-making situations [14]. These styles, seen as individual variations in decision-making patterns, were traditionally studied using established questionnaires like the General Decision-Making Scale (GDMS [14]), which outlines five distinct decision styles (rational, intuitive, avoidant, dependent, and spontaneous), or the Maximization Scale (MS [15]), which includes three key dimensions (the inclination to set high standards, the pursuit of better alternatives, and decision-making difficulty). They have also been explored in connection with various professions [16].

This study explored behavioral correlates of the influence of reframing on decision confidence and their relationship with individuals’ decision-making styles.

We achieved this by asking healthy participants to complete the RRT, a task consisting of two ecological decision-making steps. In each step, participants were presented with a critical workplace situation, asked to make a decision and to rate their confidence in their choice on a Likert scale from 1 to 5 (first confidence rating). Then, the situation was reframed by highlighting the negative consequences of their initial choice. After this reframing, participants were then asked once again to rate their confidence in their previous decision (second confidence rating). After the first step, a second step with the same confidence ratings was presented to the participants.

Confidence response scores (ranging from 1 to 5) and RTs were collected for the confidence ratings in the two steps of the task. RTs were gathered as an indirect indicator of cognitive load and the processes involved in evaluating decision-making confidence. This approach allowed us to assess participants’ cognitive effort and workload as the conditions became more challenging, while also being consistent with established research methods for measuring cognitive workload.

Furthermore, the GDMS [14] and the MS [15] were applied to profile individuals’ decision-making styles and explore potential associations between the five different GDMS decision-making styles, the MS subscales, and the behavioral response related to the decision.

We first hypothesized that, due to the reframing effect, which leads to viewing situations from a different perspective and reevaluating the choice’s outcome based on a new frame of reference, participants would exhibit significantly greater confidence in their choices prior to reframing in each step.

Secondly, we expected significantly longer RTs when participants were asked about their confidence after the reframing, as the cognitive effort required to reflect on their decision and their level of confidence within the new frame was expected to increase.

Thirdly, we expected lower confidence and increased RTs in the second compared to the first step, since the awareness of increased complexity—due to the incoming reframe—could increase participants’ reflection on their decision and impact their confidence.

Finally, given the previous results on the relation between decision-making styles and resistance to reframing [13] which showed that higher GDMS-dependent decision-making scores positively correlate with reduced resistance to this bias in a sample of professionals, we could also expect that healthy individuals with higher GMDS-dependent decision-making scores would be more vulnerable to this effect and would show lower confidence in their decision after reframing. Moreover, even a tendency to set high standards (measured with MS) was linked to decreased resistance to decision-making bias—measured with a decoy alternative task in a sample of junior professionals [13]—thus, perhaps we could expect that healthy individuals with MS High Standards would show lower confidence in their decision after reframing.

After providing this background, we will present the methodology and materials used in the experiment, report the behavioral and correlational results, and discuss the key significant findings.

## 2. Materials and Methods

### 2.1. Participants

A total of 51 healthy participants (29 males and 22 females; age range = 18–28; mean age = 22; standard deviation of age = 2.21) participated in this study. The exclusion criteria included high levels of depression and perceived stress, prior psychiatric or neurological conditions, current treatment with psychoactive medications that could impact cognitive or decision-making functions, abnormal short- or long-term memory, or low overall cognitive performance. Participants provided written informed consent and were made aware of their right to withdraw from the study at any point.

The study was approved by the Ethics Committee of the Department of Psychology of the Catholic University of the Sacred Heart, Milan, Italy (approval code: 125/24—Valutare il Decision-Making: consapevolezza e metacognizione decisionale; approval date: 23 July 2024). The study was carried out under the Declaration of Helsinki principles (2013) and the GDPR (Reg. EU 2016/679) and its ethical guidelines.

### 2.2. Procedural Steps and Experimental Task

Participants were seated in a quiet room, positioned about 80 cm from a computer screen. After providing written informed consent, they received instructions for completing the RRT on decision-making, which was administered through a web-based survey and experiment management platform (Qualtrics XM platform, version 2.61.0; Qualtrics LLC, Provo, UT, USA).

The RRT consisted of two steps. In both steps, participants were asked to relate to the situation and select the option they believed to be the most appropriate from a set of alternatives.

Decisional script: In each step, participants were faced with a decision-making scenario based on a challenging work situation that required them to make a decision. For example, in the first step, they encountered the following script:

*“You must participate together with all the executives of your company in a particularly hard decision. Due to a funding cut, you must decide whether to close some plants and lay off some employees. You have 4 factories and 6000 employees in total. Let us introduce you to some of the people who work in these establishments.”*

Following the script, participants were shown images of the four factories and the four employees mentioned. They were then asked to choose which factory they would keep open by selecting one of the four options provided.

First confidence rating (Time 1, T1): After making their decision, participants rated their confidence in their choice using a 5-point Likert scale, where 1 represented “not at all sure” and 5 represented “completely sure”. We selected a 5-point Likert scale to measure decision confidence as it offers a balance between sensitivity and ease of use for participants. Five-point scales are widely employed in research on metacognition because they provide sufficient response variability while minimizing cognitive load and potential ambiguity in rating choices [17,18].

Reframing: Then, the participants were shown the reframed version of the situation, in which they were informed that “based on the choice they have made, the other employees will lose their jobs”.

Second confidence rating (Time 2, T2): After this reframing, participants were then asked once again to rate their confidence in their decision on a Likert scale from 1 to 5, where 1 corresponded to “not at all” and 5 to “entirely sure”.

After the first step, the second step was presented to the participants and the same confidence ratings were proposed to participants (at Time 3, T3, and Time 4, T4). Confidence response scores (ranging from 1 to 5) and RTs were collected for the confidence ratings in the two steps of the task (T1, T2, T3, and T4).

**Figure 1:**
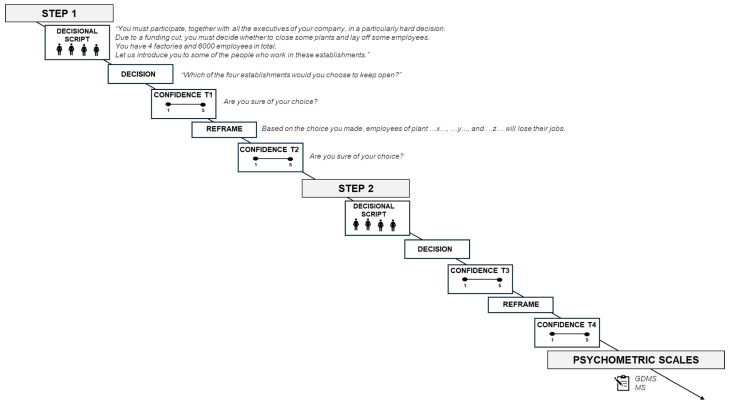
Description of the experimental task.

The GDMS and the MS were applied at the tasks’ conclusion to obtain participants’ self-report data. The experiment lasted around 20 min (Figure 1).

### 2.3. Self-Report Scales for Measuring Decision-Making Style

The Italian version of the General Decision-Making Style (GDMS) [14,19] and the Maximization Scale (MS) [15,20] were used to gather data on individuals’ decision-making styles.

The GDMS is a validated instrument designed to categorize individuals according to five distinct decision-making styles (rational, intuitive, dependent, avoidant, and spontaneous). It consists of 25 items, with each item asking participants to rate their level of agreement on a 5-point Likert scale. A person with a rational decision-making style typically makes choices after thoroughly evaluating various alternatives, relying on a detailed search for information. In contrast, someone with an intuitive style tends to make decisions based on gut feelings and attention to broader, global factors. The dependent decision-making style is characterized by a tendency to frequently seek advice and guidance from others before making decisions, while the avoidant style is marked by an inclination to delay or avoid making decisions altogether. Lastly, individuals with a spontaneous decision-making style prefer to make decisions quickly and without prolonged deliberation.

The MS is a validated questionnaire consisting of 13 items [15] requiring individuals to express their degree of agreement on a 7-step Likert scale and that allows one to measure decision-makers’ tendency (i) to hold high standards for themselves and things in general (High Standards subscale), (ii) to seek better options (Alternative Search subscale), and (iii) to encounter difficulties in making a choice (Decision Difficulty subscale).

### 2.4. Data Analysis

First, for behavioral data, two repeated-measures ANOVAs with time (4: T1, T2, T3, T4) as the within-subject factor were applied to the behavioral scores (confidence response score and RTs).

For all the ANOVA tests, when Mauchly’s test was found to be significant, the degrees of freedom were corrected using Greenhouse–Geisser epsilon where appropriate. Furthermore, the normality of the data distribution was preliminarily assessed by checking kurtosis and asymmetry indices. The normality assumption of the distribution was supported by these preliminary tests. The significance threshold was set at α < 0.05. The Bonferroni correction was applied to all pairwise comparisons computed to test simple effects for significant interaction and main effect contrasts in order to control for potential multiple-comparison biases (six comparisons for each ANOVA). The size of statistically significant effects was estimated by computing partial eta-squared (*η*^2^) indices.

Finally, Pearson correlations (*r* di Pearson), with Bonferroni corrections for multiple comparisons, were applied to the self-report data (GDMS and MS) and the behavioral scores (confidence response score and RTs).

Given that our analyses involved a manageable number of comparisons, we considered Bonferroni to be an appropriate choice to ensure strict control of Type I errors, despite its conservative nature [21].

## 3. Results

### 3.1. Behavioral Results

**Figure 2:**
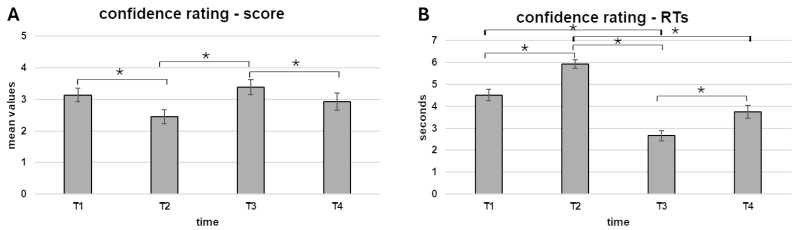
(**A**,**B**). Behavioral results. (**A**) The bar graph shows a higher confidence score in the choice in T1 compared to T2, and in T3 compared to T2 and T4. (**B**) The bar chart displays higher RTs for T1 compared to T3, as well as higher RTs in T2 compared to T1, T3, and T4. Also, higher RTs were found in T4 compared to T3. Bars represent standard errors. Stars mark statistically significant differences (*p* ≤ 0.005).

The ANOVAs for the behavioral confidence score showed a main effect for time (*F* [3, 81] = 9.39, *p* < 0.001, *η*^2^ = 0.071). Pairwise comparisons revealed higher confidence in the choice in T1 compared to T2 (*p* = 0.007), and in T3 compared to T2 (*p* < 0.001) and T4 (*p* = 0.009) (Figure 2A).

Secondly, a main effect for time (*F* [3, 93] = 46.3, *p* < 0.001, *η*^2^ = 0.422) was also found for the RTs. Pairwise comparisons revealed higher RTs for T1 compared to T3 (*p* < 0.001), as well as higher RTs in T2 compared to T1 (*p* < 0.001), T3 (*p* < 0.001) and T4 (*p* < 0.001). Also, higher RTs were found in T4 compared to T3 (*p* < 0.001) (Figure 2B).

### 3.2. Correlational Results Between Psychometric and Response Scores

**Figure 3:**
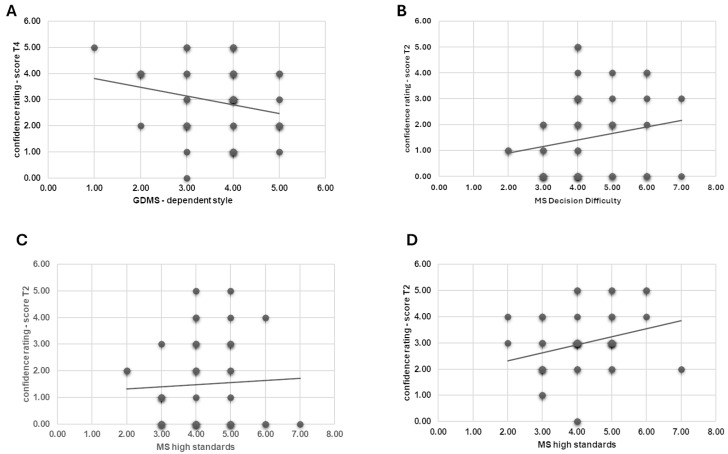
(**A**–**D**). Correlational results for psychometric measures and response score. The scatterplots represent a significant (**A**) negative correlation between GDMS-dependent style and confidence response score in T4; (**B**) positive correlation between MS Decision Difficulty and confidence response scores in T2; (**C**) positive correlation between MS High Standard and confidence response scores in T2; (**D**) positive correlation between MS High Standard and confidence response scores in T1.

First, correlational analysis revealed a negative correlation between GDMS-dependent style and confidence response score in T4 (*r* = −0.289, *p* = 0.049) (Figure 3A).

Secondly, a positive correlation was found between MS Decision Difficulty and confidence response scores in T2 (*r* = 0.450, *p* = 0.016) (Figure 3B), as well as between MS High Standard and confidence response scores in T2 (*r* = 0.023, *p* = 0.031) (Figure 3C).

Thirdly, a positive correlation was found between MS High Standard and confidence response scores in T1 (*r* = 0.300, *p* = 0.043) (Figure 3D). No other significant correlations were found.

### 3.3. Correlational Results Between Psychometric Measures and RTs

**Figure 4:**
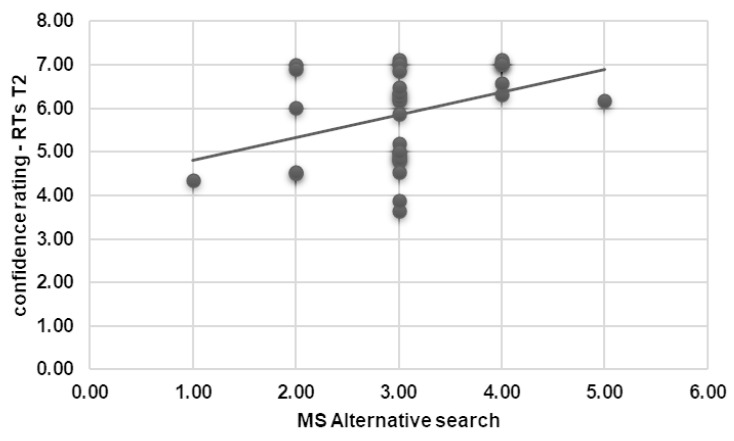
Correlational results for psychometric measures and RTs. The scatterplot displays a significant positive correlation between MS Alternative Search and the RTs in T2.

A positive correlation was also found between MS Alternative Search and the RTs in T2 (*r* = 0.376, *p* = 0.034) (Figure 4). No other significant correlations were found.

## 4. Discussion

This study explored the behavioral correlations of the influence of reframing on decision confidence and their relationship with individuals’ decision-making styles in a sample of healthy adults.

First, a reframing effect was found: participants were significantly more confident in their choice before the reframing in each step. Also, as a marker of increased cognitive load, significantly higher RTs were found when participants were asked about their confidence after the reframing in both steps. Secondly, regarding the steps of the task, participants appeared more confident and quicker in their responses, on average, in the second step than in the first. Finally, correlational results revealed significant relationships between psychometric measures of decision-making styles (GDMS, MS) and participants’ confidence ratings and RTs, particularly after the reframing. These findings will be discussed in detail below.

Starting with the behavioral results, firstly, a reframing effect was found: participants were significantly more confident in their choice before the reframing in each step. This result aligns with our first hypothesis that reframing alters the perspective from which individuals evaluate their initial decision [2], potentially introducing doubt or cognitive dissonance regarding the consequences of their choice. The reduction in confidence after reframing suggests that the task’s reframing was effective in challenging participants’ initial assessments, requiring them to reevaluate the implications of their decisions. This further demonstrates that reframing not only impacts the decision but also the level of confidence in making the decision. Furthermore, as a marker of increased cognitive load, significantly higher RTs were found when participants were asked about their confidence after reframing for both steps. In line with our second hypothesis, these results underscore the cognitive load induced by reframing, as participants had to reconcile the new frame with their prior decision. A possible alternative explanation could be that emotional reactions to reframing (e.g., frustration, doubt) or the uncertainty introduced by a change in perspective may also influence RTs by causing participants to pause and deliberate more before making a decision. Future research could investigate these factors directly, perhaps through additional measures of emotional states or uncertainty, to better distinguish between cognitive load and emotional or judgmental hesitations.

Differently from what we hypothesized in our third hypothesis, participants appeared more confident and quicker in their response on average in the second step than in the first step. This difference may be explained by the fact that participants were better able to process and integrate information in the second step, potentially due to familiarity gained from the first step or increased awareness about the dynamic of the task. As an alternative explanation, it might be that the specific content or framing of the second step may have been perceived as less ambiguous or emotionally demanding, fostering greater overall confidence in the choice. To gain a deeper understanding of this effect and determine whether it is influenced by experience, future studies could incorporate a larger variety of scenarios.

Also, compared to the first step, at the beginning of the second step, participants showed higher confidence levels and quicker RTs. These findings could likely reflect the greater cognitive effort required at the onset of the task, when participants were unfamiliar with the decision-making paradigm. And reduced RTs might suggest that participants were more efficient in processing information and making confidence judgments in the second step, likely due to task habituation.

Moreover, significantly higher RTs were identified the first time participants were presented with the reframe in the first step compared to all other confidence-rating times. This may suggest that the first reframing instance imposed the greatest cognitive demand, potentially due to participants’ initial exposure to this shift in perspective. Or, alternatively, it could be due to a sort of novelty effect introduced by the reframe. Subsequent decreases in RTs may reflect a learning effect or desensitization to the reframing strategy, allowing participants to adapt their confidence in the decision-making process more efficiently.

Regarding correlational outcomes, the findings of this study provided insight into how individual differences in decision-making styles and cognitive traits are linked to responses to reframed scenarios.

Firstly, we observed a negative correlation between the GDMS-dependent decision-making style and confidence in the decision after the second reframe (in T4). This suggests that individuals with a dependent decision-making style—those who seek external input and validation—feel less confident in their decisions at the end of the second step, after the reframing. This lack of confidence may stem from their reliance on external support [22], which could be disrupted by repeated reframes that challenge initial assumptions or decisions.

Secondly, individuals with high MS Decision Difficulty scores showed a positive correlation with confidence ratings after the first reframe (in T2). Despite the overall trend of reduced confidence among participants in the first step after the first reframing in T2, those who tend to encounter difficulties in decision-making (as measured by Nenkov and colleagues’ subscale [15]) displayed greater certainty in their decisions in that specific condition. This paradox may indicate a compensatory effect, where individuals with high decision difficulty may have invested more cognitive effort to maintain confidence when confronted with reframed information.

Thirdly, a positive correlation was found between MS High Standards and confidence ratings after the first reframe (in T2). As well as for individuals with high MS Decision Difficulty scores, participants with high standards in decision-making remained more certain of their choices in T2, resisting the destabilizing effect of the reframe. This suggests that adherence to personal standards may provide a psychological anchor, enabling these individuals to maintain their confidence even when faced with challenging or altered perspectives.

The same positive correlation was also observed between MS High Standards and confidence in the first confidence ratings (in T1). This indicates that individuals with high standards tend to feel more confident in their initial decisions, a trend that persists after the reframe in the first step (in T2). Their strong initial confidence likely stems from their rigorous decision-making process, which makes them more resistant to external influences like reframing. These results partially contradict our initial hypothesis, which posited that healthy individuals with high MS standards might exhibit lower confidence in their decisions following reframing. In a previous study, we observed that junior professionals with high MS standards demonstrated reduced resistance to decision-making biases [13]. However, the differing results in this study may be explained by variations in the task and the characteristics of the sample. Here, the MS High Standards decision-making style appears to be the most protective factor since individuals with this style showed greater confidence in their decisions both before and after reframing.

Fourthly, a positive correlation was identified between MS Alternative Search and RTs after the first reframe (T2). Participants with higher scores on the MS Alternative Search scale—those who actively seek alternative options—tended to take longer to decide in T2. This aligns with the finding that T2, the first reframed scenario, is generally the point where participants exhibit the greatest hesitation and difficulty. For individuals who habitually evaluate multiple alternatives, the cognitive demands of processing the reframed context likely amplify their decision time.

To the best of our knowledge, this is the first time behavioral correlates of the influence of reframing on decision confidence and their relationship with individuals’ decision-making styles in a sample of healthy adults have been evaluated. The scenarios adopted in this study concern ecological conditions related to the organizational world, particularly cases of dismissal; however, the same experimental impact can be applied to other significant decisions, and to various practical contexts, such as the medical or political fields. Future research could address these limitations by incorporating a broader range of scenarios to further investigate the observed dynamics, more diverse populations, and the integration of neuroscientific techniques, such as the electroencephalogram, which have the potential to provide deeper and more precise insights into cognitive load. Also, as the last limitation, we acknowledge that the effects of neutral or positive reframing may differ from the current results, and future research should explore these variations to determine whether the observed shifts in confidence are specific to negative reframing or part of a broader reframing effect. Although our study does not include a control group with neutral or positive reframing, our findings lay a solid foundation for investigating these additional conditions in future work.

## 5. Conclusions

This study investigated how reframing influences decision confidence and its interaction with individual decision-making styles, providing valuable contributions to the understanding of decision-making processes and their potential applications across various applied contexts.

The results indicate that reframing significantly lowers participants’ confidence, particularly when encountered for the first time, demonstrating its ability to challenge initial decisions. Additionally, the observed increase in response times following reframing suggests that shifting perspectives introduces greater cognitive complexity, shedding light on the dynamics of the decision-making process. Correlational analyses revealed the relationship between individual decision-making styles and behavioral responses to reframed situations. While certain traits, such as a dependent decision-making style, lead to decreased confidence in reframed scenarios, others—such as high personal standards and decision difficulty—appear to strengthen it. Likewise, individuals who engage in extensive alternative searching (MS Alternative Search) exhibit longer response times, suggesting that reframed decision contexts demand increased cognitive effort. These findings highlight how individual differences can serve as either protective or risk factors in susceptibility to covert influence tactics and cognitive biases in decision-making.

Future research could explore several avenues to deepen our understanding of these effects. For instance, investigating how repeated exposure to reframing influences confidence over time could provide insights into potential adaptation mechanisms. Additionally, examining the neural correlates of reframing effects using neuroimaging techniques could reveal the underlying cognitive processes involved in shifting perspectives. It would also be valuable to explore these dynamics in real-world decision-making contexts, such as financial or medical decision-making, to assess the broader applicability of these findings. Lastly, studying interventions that mitigate the confidence-reducing effects of reframing could offer practical strategies to enhance decision resilience in high-stakes environments.

## Data Availability

The data presented in this study are available on request from the corresponding author due to ethical reasons for sensitive personal data protection (requests will be evaluated according to the GDPR—Reg. UE 2016/679 and its ethical guidelines).
